# Genome-wide association study identifies candidate genes related to oleic acid content in soybean seeds

**DOI:** 10.1186/s12870-020-02607-w

**Published:** 2020-08-28

**Authors:** Xiaoyi Liu, Di Qin, Angela Piersanti, Qi Zhang, Cristina Miceli, Piwu Wang

**Affiliations:** 1 Research Center of Integrative Medicine, School of Basic Medical Sciences, Guangzhou University of Chinese Medicine, Guangzhou, 510006 Guangdong PR China; 2 School of Biosciences and Veterinary Medicine, University of Camerino, Camerino, MC Italy; 3 Biotechnology Center of Jilin Agricultural University, Jilin Agricultural University, Changchun, 130118 PR China

**Keywords:** Soybean, Oleic acid, SNPs, Genome-wide association study, Oleic acid-associated genes

## Abstract

**Background:**

Soybean oil is a complex mixture of five fatty acids (palmitic, stearic, oleic, linoleic, and linolenic). Soybean oil with a high oleic acid content is desirable because this monounsaturated fatty acid improves the oxidative stability of the oil. To investigate the genetic architecture of oleic acid in soybean seeds, 260 soybean germplasms from Northeast China were collected as natural populations. A genome-wide association study (GWAS) was conducted on a panel of 260 germplasm resources.

**Results:**

Phenotypic identification results showed that the oleic acid content varied from 8.2 to 35.0%. A total of 2,311,337 single-nucleotide polymorphism (SNP) markers were obtained. GWAS analysis showed that there were many genes related to oleic acid content with a contribution rate of 7%. The candidate genes *Glyma.11G229600.1* on chromosome 11 and *Glyma.04G102900.1* on chromosome 4 were detected in a 2-year-long GWAS. The candidate gene *Glyma.11G229600.1* showed a positive correlation with the oleic acid content, and the correlation coefficient was 0.980, while *Glyma.04G102900.1* showed a negative correlation, with a coefficient of − 0.964.

**Conclusions:**

*Glyma.04G102900.1* on chromosome 4 and *Glyma.11G229600.1* on chromosome 11 were detected in both analyses (2018 and 2019). *Glyma.04G102900.1* and *Glyma.11G229600.1* are new key candidate genes related to oleic acid in soybean seeds. These results will be useful for high-oleic soybean breeding.

## Background

Soybean [*Glycine max* (L.) Merrill] originated in China and has been cultivated for more than 3000 years [1]. Soybean oil accounts for 20–25% of the total global fat and oil production and 30–35% of the total edible vegetable oil production [2]. In China, soybean oil is an important constituent of the diet, and it is considered a major factor in the maintenance of a healthy population. Soybean oil is a complex mixture of five fatty acids (palmitic, stearic, oleic, linoleic, and linolenic), all of which have different melting points, oxidative stabilities, and chemical functionalities [3].

The fatty acid composition of soybean oil is approximately 5 to 11% linolenic acid, 43 to 56% linoleic acid, 15 to 33% oleic acid, and 11 to 26% saturated acids [4]. Palmitic acid and stearic acid are saturated fatty acids that constitute 15% of soybean oil. In recent times, there has been a running debate, mainly in mainstream literature, regarding the effects of palmitic acid and stearic acid consumption on heart function, especially in the development of coronary artery disease [5, 6]. Linolenic and linoleic acids are polyunsaturated fatty acids that constitute 80% of soybean oil. Linolenic acid is needed for normal human growth and development and can lower the cholesterol content in blood, but this acid is not resistant to high temperatures. Atmospheric oxygen and ultraviolet rays can oxidize linolenic acid, resulting in the odour of soybean oil, which lowers the nutritional value of soybean oil. Oleic acid is a monounsaturated fatty acid, and soybean seeds with high oleic acid content also exhibited reduction in or elimination of chemical hydrogenation processes, reducing the cost of soybean oil processing [7]. The cultivation of soybean varieties with high oleic acid content has become an important goal of high-quality soybean breeding [8].

Genome-wide association analysis (GWAS) presents a powerful tool to connect this trait with the underlying genetics. With the rapid development of next-generation sequencing technology, GWAS has been successfully applied to plants such as rice and *Arabidopsis* [9, 10]. A large number of genetic variations associated with complex traits have been identified by the GWAS method [11]. In soybean, GWAS was performed to identify quantitative trait loci (QTLs) controlling seed oil concentration in 298 soybean germplasm accessions that exhibited a wide range of seed protein and oil content [12]. A soybean breeding germplasm population (279 lines) was established to perform a GWAS, and 8 QTLs were found that explained phenotypic variances ranging from 6.3 to 26.3% [13]. These results demonstrated that the use of GWAS with specially designed mapping populations is effective in uncovering the basis of key agronomic traits.

Scientists have successfully used the GWAS method to obtain a large number of candidate genes [14–16]. However, after the discovery of new candidate genes, verifying their biological function becomes a hot topic of research; RNA interference technology, the establishment and application of biochips, real-time fluorescent quantitative PCR technology (qRT-PCR), and gene editing technology provide, theoretically, a basis for candidate gene function verification. In this study, we selected two key candidate genes and measured their expression in four different tissues (root, stem, leaf and seed) of 14 diverse soybean lines appropriately selected using qRT-PCR.

Specifically, 260 soybean germplasms from Northeast China (Heilongjiang Province, Jilin Province and Liaoning Province) were collected as natural populations. The soybean lines were planted in the field of Jilin Agricultural University from 2018 to 2019. The fatty acid content in the soybean seeds as determined by a NIRS DS 2500 instrument after harvest. Specific-locus amplified fragment sequencing (SLAF-seq) technology was used to sequence the genomes of the 260 soybean materials, and GWAS was used to screen candidate genes related to soybean oleic acid content.

## Results

### Phenotypic identification of oleic acid content in soybean seeds

**Fig. 1:**
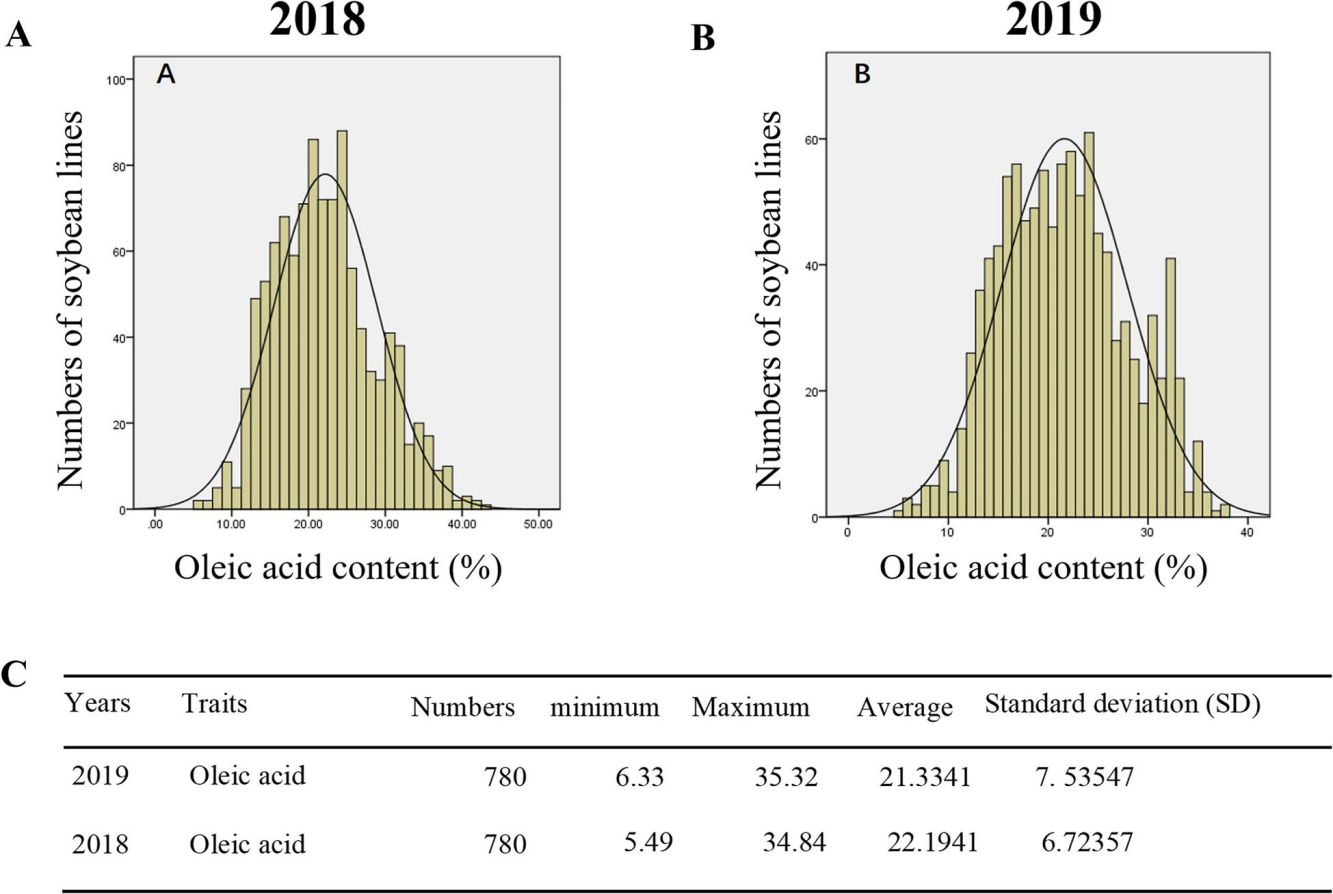
Frequency distribution of oleic acid content in soybean seeds in 2018 and 2019. The X-axis on the graph shows the range of content of oleic acid. The Y-axis is the number of soybean lines. The distributions of oleic acid content in 2018 (**a**) and in 2019 (**b**) resemble the bell-shaped curve for a normal distribution. (**c**) Statistical analysis of oleic acid content in soybean seeds from 2018 to 2019

From 2018 to 2019, the oleic acid content of each soybean line was analysed by SPSS 22.0 software. The oleic acid content of the seeds approached the normal distribution (Fig. 1a, b). The standard deviation (SD) of the oleic acid content of soybean seeds was 6.7 in 2018 and 7.5 in 2019 (Fig. 1c). The normal distribution is a probability function that describes how the values of a variable are distributed. It is a symmetric distribution where most of the observations cluster around the central peak, and the probabilities for values farther away from the mean taper off equally in both directions. The results also indicated that the oleic acid content in different soybean lines is significantly different, and the distribution of the oleic acid content in soybean seeds is continuous, which is consistent with the genetic law of quantitative traits. The oleic acid content in the soybean line q070 was 35.32% in 2019 and 34.84% in 2018, which was the highest among all the soybean lines. The oleic acid content in the soybean line q024 was 5.49% in 2018 and 6.33% in 2019, which was the lowest among all the soybean lines.

### Soybean fatty acid correlation analysis and heritability calculation

**Fig. 2:**
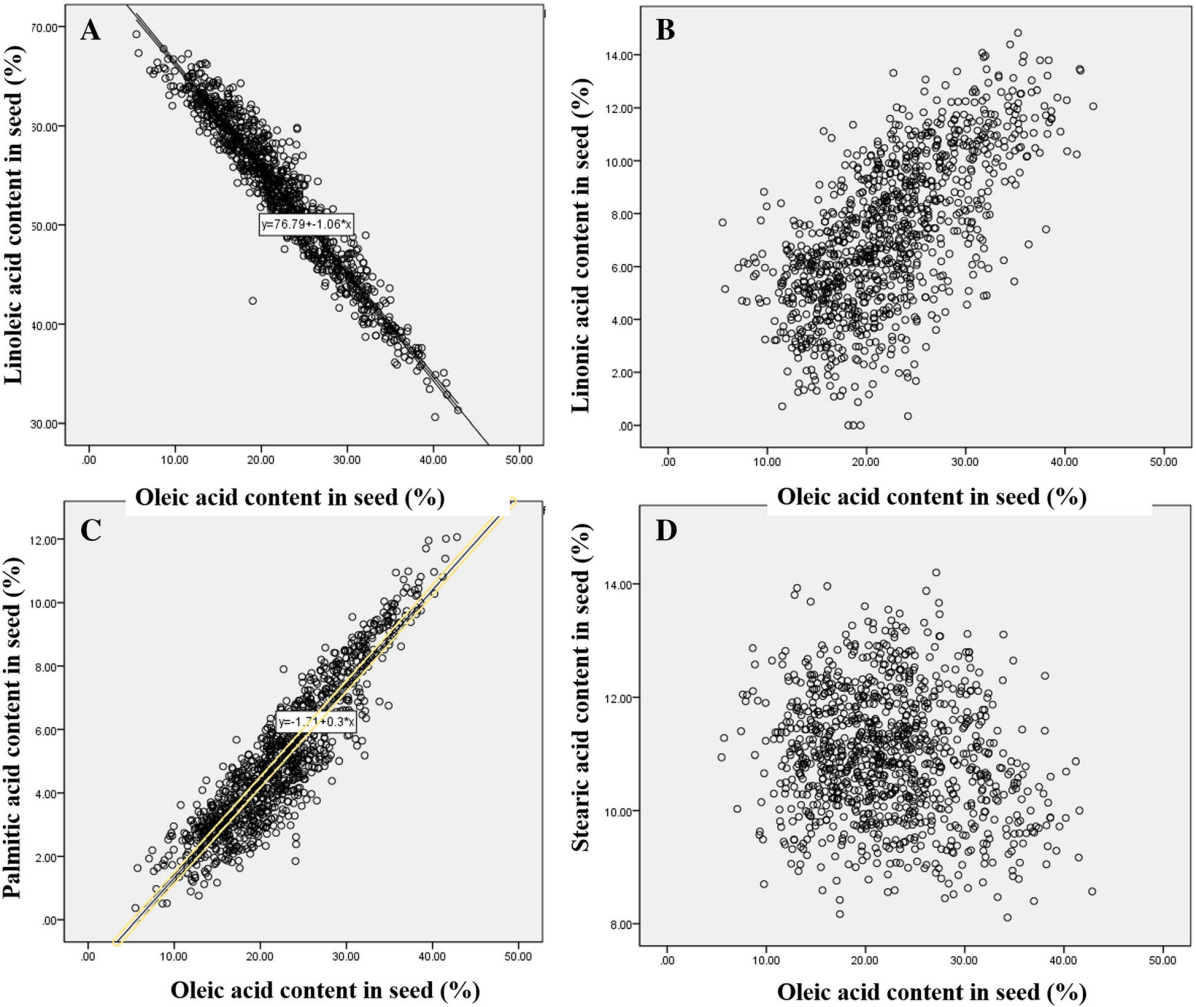
The relationship between oleic acid and other fatty acids. **a** Association between percentages of oleic acid and linoleic acid, *y = 1.06x + 76.79*, is a linear equation, where y represents linoleic acid content and x represents oleic acid content. **b** Association between percentages of oleic acid and linolenic acid. **c** Association between percentages of oleic acid and palmitic acid, *y = 0.3x + 1.71*, is a linear equation, where *y* represents palmitic acid content and *x* represents oleic acid content. d Association between percentages of oleic acid and stearic acid

The multiple linear regression model was used to analyse the relationship between different fatty acids. According to the regression coefficients of the standardized multiple linear regression model, the soybean oleic acid content is significantly negatively correlated with the linoleic acid content (Fig. 2). The correlation coefficient between oleic acid and linoleic acid is − 0.660 (Table 1). There was a significant positive correlation between soybean oleic acid content and palmitic acid content, with a correlation coefficient of 0.581. The heritability of the five fatty acids in 260 soybean lines was different, and the heritability of oleic acid was 0.652 (Additional file 1: Table S1).

**Table 1:** Correlations of the relative oleic acid content in soybean

		Oleic acid	Linoleic acid	Linolenic acid	Palmitic acid	Stearic acid
Oleic acid	Pearson correlation	1	−0.660^b^	0.332^a^	0.581^b^	−0.254^b^
Linoleic acid	Pearson correlation	−0.660^b^	1	0.571^b^	0.051^b^	−0.893^b^
Linolenic acid	Pearson correlation	0.332^a^	0.571^b^	1	0.447^b^	−0.473^b^
Palmitic acid	Pearson correlation	0.581^b^	0.051^b^	0.447^b^	1	−0.043^b^
Stearic acid	Pearson correlation	−0.254^a^	−0.893^b^	− 0.473^b^	−0.043^b^	1

**Note:** ^a^ indicates significance at 0.05, ^b^ indicates significance at 0.01

### SNP genotyping and SNP annotation

**Fig. 3:**
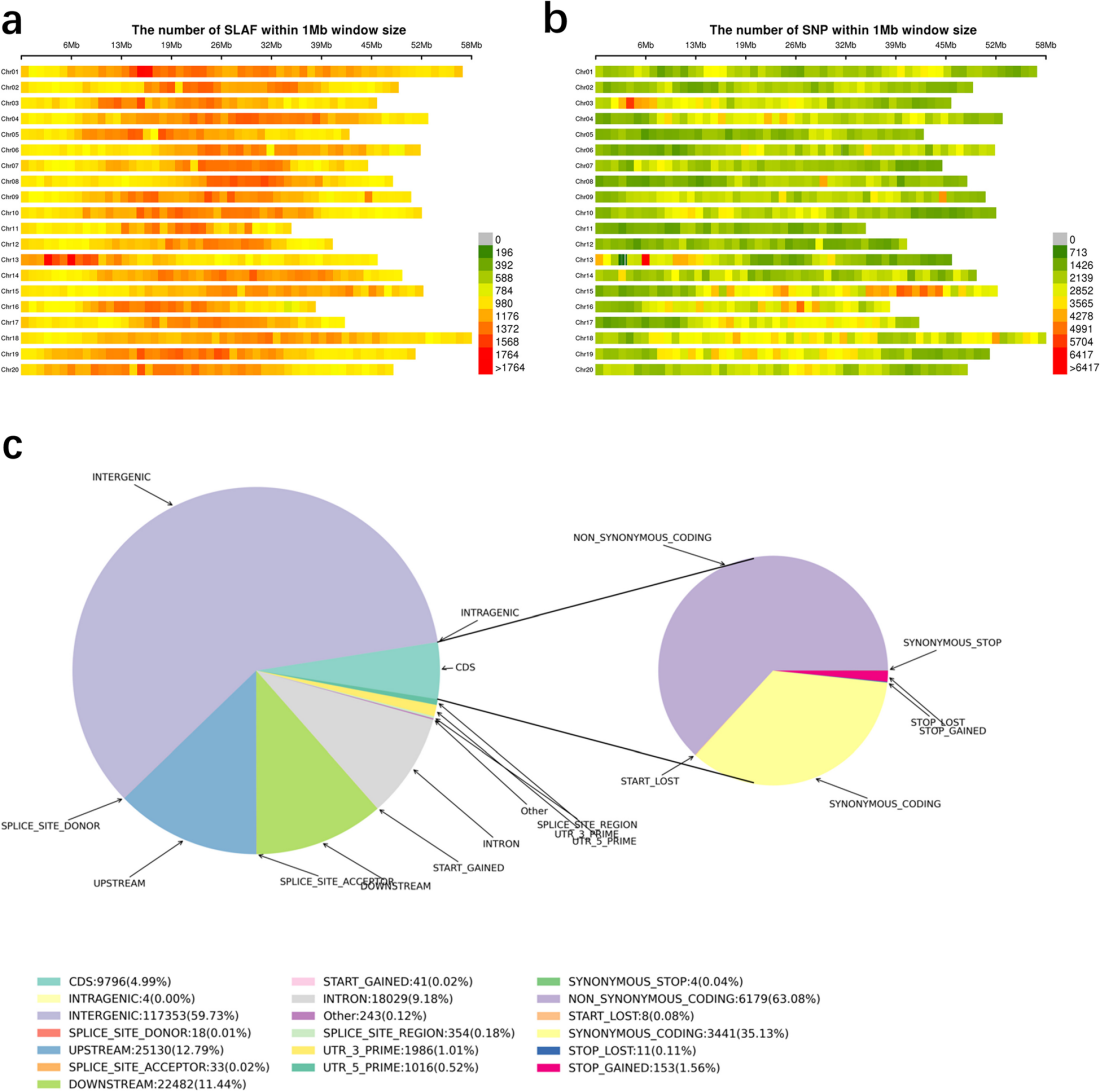
Genotyping and SNP Annotation. The abscissa is the length of the chromosome. Each band represents one chromosome. The genome is divided according to the size of 1 Mb. **a** Distribution Map of SLAF on chromosomes. The SLAF label is concentrated in the red area of map. **b** Distribution Map of SNPs on different chromosome. The red color represents more than 5706 SNP markers in the region, the yellow color represents a number of SNP markers between 2852 and 3565. **c** Pie chart of SNPs annotation. Left panel: SNPs percentages generally associated to genes. Right panel: SNPs percentages in coding regions

In our experiments, SLAF-seq technology was used to sequence soybean genomic DNA. A total of 1,102,987 SLAF tags and 2,311,337 SNP markers were obtained (Fig. 3a, b). The results of SNP distribution on chromosomes are shown in Fig. 3b. According to the location information for SNP loci in the reference genome (CDS regions, gene regions or intergenic regions), the mutated loci (non-synonymous mutations) were predicted. More than 50% of the SNPs were located in the intergenic regions (stretches of DNA sequences located between genes). Ten percent of the SNP markers were located in the upstream region of genes, and 10% of the SNPs were located in the downstream region of genes. A total of 4.99% of the SNP loci were located in the protein-coding regions. Nine percent of the SNP markers were located in introns (Fig. 3c).

### Phylogenetic analysis, genetic structure analysis, principal components analysis (PCA)

**Fig. 4:**
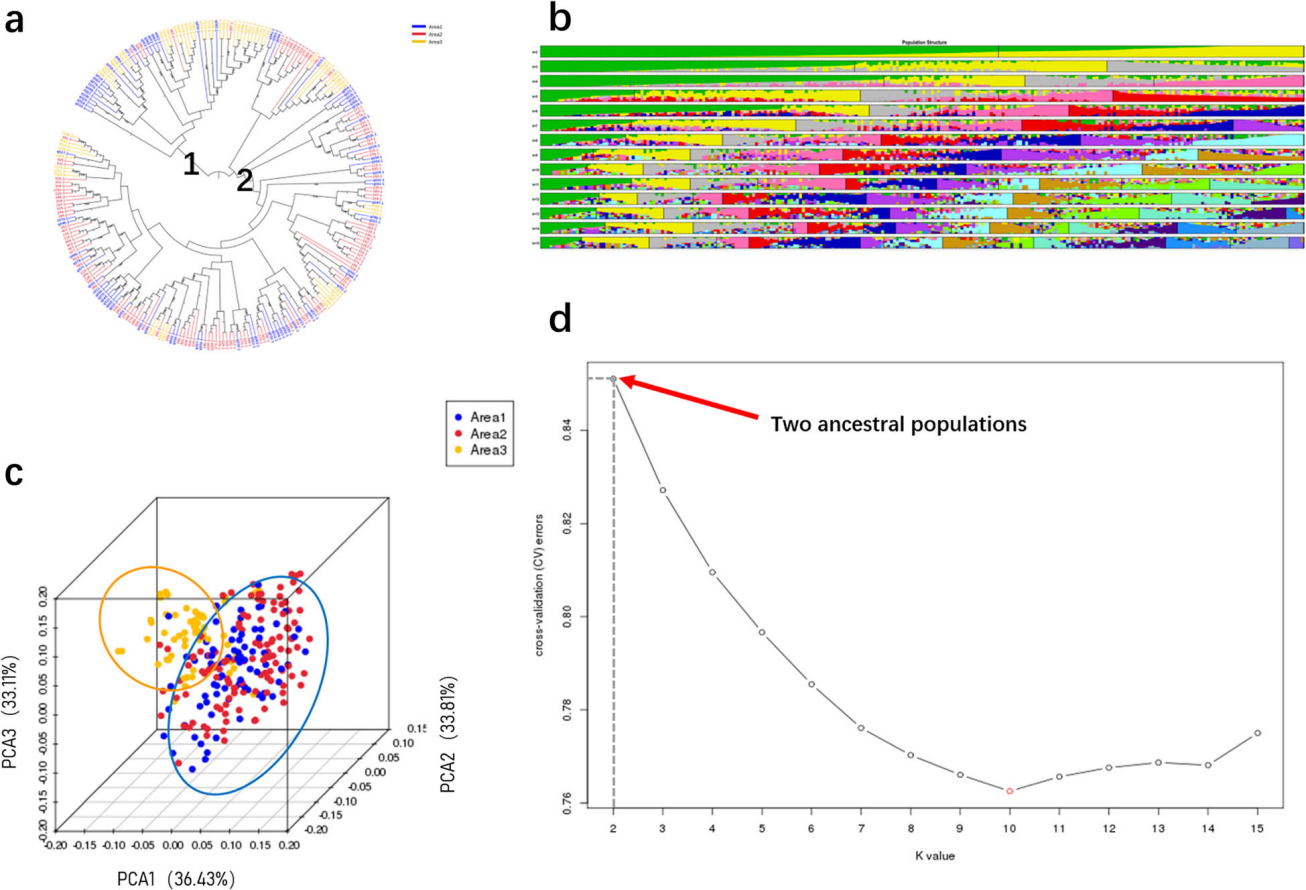
Group Structure of 260 soybean lines **a** Phylogenetic tree of 260 soybean lines. **b** Clustering analysis when the number of subgroups is in the range 2–13, the colours represent separate groups. **c** Three-dimensional score plot (PC1, PC2, PC3) to discriminate between soybeans lines from three provinces of China. **d** Diagram showing the value of 260 samples based on clustering from 1 to 15; Cross validation error rate for each K value of 1–15, X-axis is K value 1–15, Y-axis is cross-validation errors. Aea1 represents the soybean lines from Jilin province, Area2 represents the soybean lines from Heilongjiang province, Area3 represents the soybean lines from Liaoning province

From the phylogenetic tree, it can be concluded that the soybean lines originated from two large branches. This result suggests that the 260 soybean lines were from the same ancestor. However, in the process of evolution, they evolved in two directions (Fig. 4a).

Population structure analysis can quantify the number of ancestors of the studied population and infer the source of each sample. It is a cluster analysis method that is currently widely applied and is helpful for understanding the evolutionary process of materials based on SNPs. This experiment was used to analyse the soybean population structure. For the study population, the number of subpopulations preset in this trial was 15 (Fig. 4b). We analysed the data with EIGENSTRAT for the study of 260 soybean lines. It was concluded that the samples that we collected can be represented as an admixture of two ancestral populations (Fig. 4d).

Based on the difference in SNPs, we performed principal component analysis by using EIGENSOFT software for clustering of the 260 soybean materials. The PCA results showed that the 260 soybean lines clustered together in two subgroups (Additional file 2: Fig. S1). PC1, PC2 and PC3 accounted for 36.43, 33.82 and 33.12%, respectively (Fig. 4c).

### Genome-wide association study (GWAS) for seed oleic acid content

**Fig. 5:**
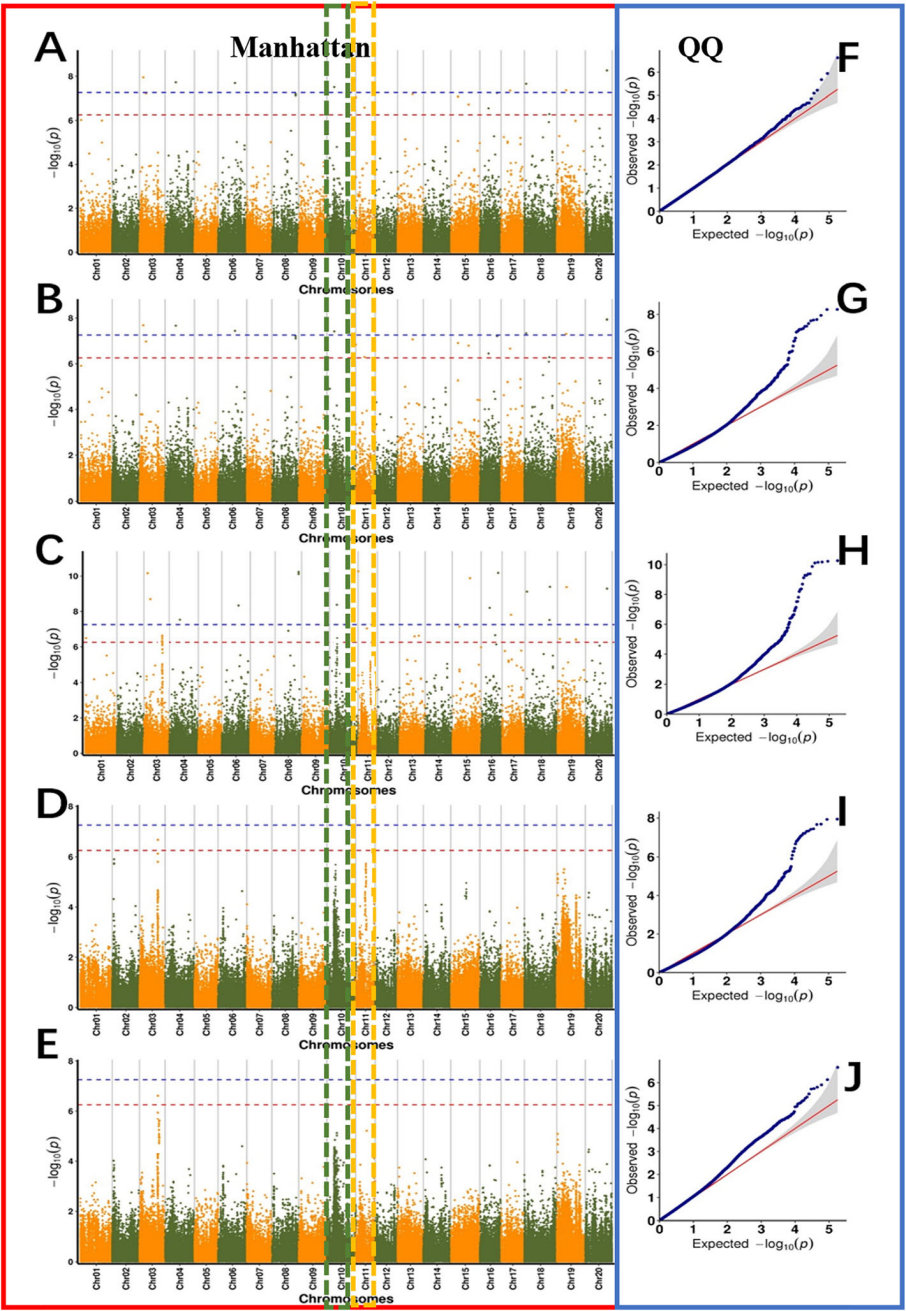
Genome-wide Manhattan plots of associations to oleic acid content in the 2018 analysis. In the left panel, the X-axis indicates the SNPs along each chromosome; the Y-axis is the −log 10 (*P*-value) for the association, the threshold value was set at –log(*p*) > 6.20 (red) and –log(*p*) > 7.20 (blue). **a** to **e** are GWAS results based on the cmlm model, EmMax model, fastlmm model, GLM, and MLM are in F to J, respectively. **e** GWAS result based on the MLM model. Q-Q plots for oleic acid using cmlm model (**f**), EmMax model (**g**), fastlmm model (**h**), GLM (**I**), MLM (**j**). The grey area represents the 95% concentration band. Each dot represents a SNP

**Fig. 6:**
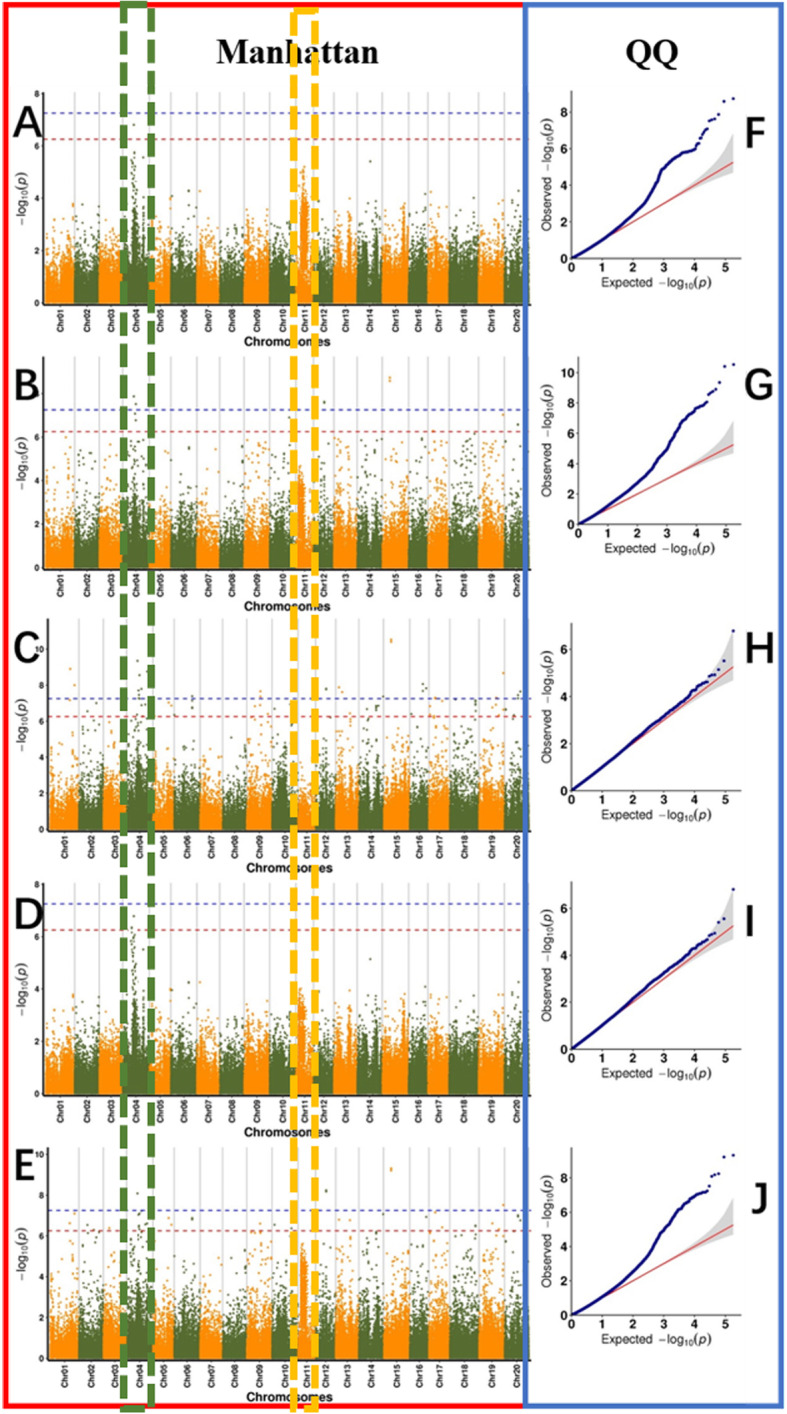
Genome-wide Manhattan plots of associations for oleic acid content for 2019 analysis. In the left panel, the X-axis indicates the SNPs along each chromosome; the Y-axis is the −log 10 (P-value) for the association, the threshold value was set at –log(*p*) > 6.20 (red) and –log(*p*) > 7.20 (blue). **a** GWAS result based on the cmlm model, **b** GWAS result based on the EmMax model, **c** GWAS result based on the fastlmm model, **d** GWAS result based on the GLM model, **e** GWAS result based on the MLM model. Q-Q plots for oleic acid using cmlm model (**f**), EmMax model (**g**), fastlmm model (**h**), GLM (**I**), MLM (**j**). The grey area represents the 95% concentration band. Each dot represents a SNP

**Fig. 7:**
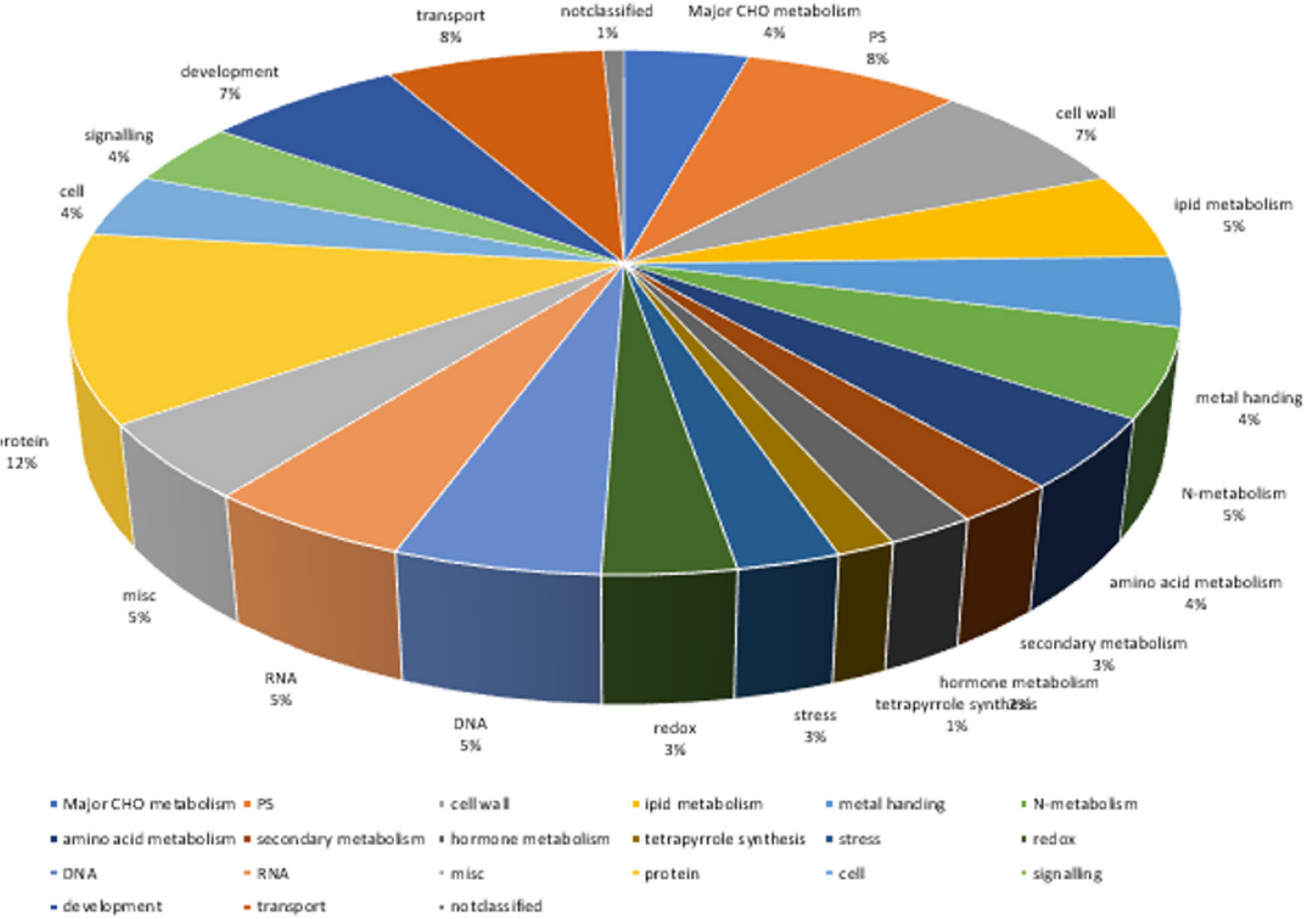
Distribution Map of Candidate Genes in the different functional process

Based on the oleic acid content of 260 soybean lines, TASSEL software (Glm model, mlm model, cmlm model), fastlmmc software, and Emmax software were used for the GWAS. SNP markers significantly correlated with the oleic acid content of soybean seeds were detected, and the linkage disequilibrium (LD) distance was set to 8.9 kb. The Manhattan and QQ (quantile-quantile) diagrams for the oleic acid content in 2018 and 2019 are shown in Fig. 5 and Fig. 6. A search was performed for SNP markers that were significantly correlated with the oleic acid content of soybean seeds (Additional file 3: Table S2). In this study, we evaluated genome-wide LD in 260 accessions and found that the LD (R^2^) values decayed to half of the maximum value within 9.5 kb (Additional file 4: Fig. S2). Using 9.7 kb as the linkage disequilibrium attenuation distance, candidate genes related to soybean oleic acid traits were screened within the LD distance. In 2018, 21 candidate genes related to soybean oleic acid content were screened using genome-wide association analysis (Table 2). In 2019, 8 candidate genes were screened by genome-wide association analysis (Table. 2). Based on GO terms, the functions of the genes were as follows: 1. major CHO metabolism (GO:0004527), 2. cell wall (GO:0009058), 3. lipid metabolism (GO:0005737), 4. metal ion binding (GO:0046872), 5. N metabolism (GO:0006499), 6. amino acid metabolism (GO:0008152), 7. secondary metabolism (GO:0016491), 8. stress (GO:0034976), 9. redox (ECO:0000313), 10. misc. (GO:0004526), 11. protein (GO:0004674), 12. cell (GO:0051510), 13. signalling (GO:0009862), 14. development (GO:0007275), and 15. transport (GO:0003712). The functional distribution of candidate genes is shown in Fig. 7. The first promising candidate gene, *Glyma.11G229600.1,* located on chromosome 11, was detected by GWAS during 2018 and 2019. Its function was not annotated in the soybean database. According to Swissprot annotation, the *Glyma.11G229600.1* gene belongs to the plant BAG protein family. The second promising candidate gene, *Glyma.04G102900.1,* located on chromosome 4, was also detected by GWAS in the 2 years. Again, there is no report regarding the function of this candidate gene in the soybean database, according to Swissprot annotation. A similar gene in *Arabidopsis* belongs to the plant GRAS protein family.

**Table 2:** Correlation between relative gene expression and oleic acid content in soybean

Year	Chromosome	Gene	Predicted function	Length	Contribution rate
2018	Chr03	*Glyma.03G054100.1*	3PREDICTED: *Glycine max* TMV resistance protein N-like (LOC100805036), transcript variant X3, mRNA	687	0.10
		*Glyma.03G168200.3*	3PREDICTED: Glycine max pleiotropic drug resistance protein 1-like (LOC100791601), mRNA	4662	0.07
	Chr04	*Glyma.04G191100.1*	3PREDICTED: Glycine max probable pectate lyase 18-like (LOC100814679), mRNA	1657	0.32
		*Glyma.04G102900.1*		2522	0.43
		*Glyma.04G203200.1*	3PREDICTED: Glycine max respiratory burst oxidase homolog protein C-like (LOC100800248), mRNA	2440	0.08
	Chr05	*Glyma.05G155300.1*	3PREDICTED: Glycine max ATP carrier protein 2, chloroplastic-like (LOC100797684), mRNA	1655	0.11
	Chr07	*Glyma.07G033100.1*	3PREDICTED: Glycine max ADP,ATP carrier protein 1, chloroplastic-like (LOC100793284), mRNA	2317	0.12
		*Glyma.07G089000.1*	3PREDICTED: Glycine max VIN3-like protein 1-like (LOC100780157), transcript variant X2, mRNA	2756	0.10
	Chr08	*Glyma.08G019700.1*	3PREDICTED: Glycine max calcium-dependent protein kinase 3-like (LOC100777096), transcript variant 1, mRNA	1877	0.16
		*Glyma.08G185000.2*	3PREDICTED: Glycine max probable plastid-lipid-associated protein 4, chloroplastic-like (LOC100803367), transcript variant 1, mRNA	979	0.15
	Chr11	*Glyma.11G229600.1*	3PREDICTED: Glycine max DNA replication complex BAG protein, transcript variant 2, mRNA,	1257	0.47
	Chr13	*Glyma.13G163400.1*	3PREDICTED: Glycine max protein S-acyltransferase 24-like (LOC100777470), misc_RNA	2490	0.32
	Chr14	*Glyma.14G045100.1*	3PREDICTED: Glycine max abscisic-aldehyde oxidase-like (LOC100812604), mRNA	4517	0.22
	Chr15	*Glyma.15G117700.1*	3PREDICTED: Glycine max uncharacterized LOC102666654 (LOC102666654), mRNA	693	0.17
		*Glyma.15G120100.1*	3PREDICTED: Glycine max tRNA methyltransferase 10 homolog A-like (LOC100779099), mRNA	1337	0.10
		*Glyma.15G120200.2*	3PREDICTED: Glycine max uncharacterized LOC102665381 (LOC102665381), mRNA	1227	0.08
		*Glyma.15G127500.1*	3PREDICTED: Glycine max polygalacturonase-like (LOC100785701), mRNA	1551	0.10
		*Glyma.15G201700.1*	3PREDICTED: Glycine max uncharacterized LOC100814752 (LOC100814752), mRNA	1945	0.11
		*Glyma.15G210100.3*	3PREDICTED: Glycine max alpha,alpha-trehalose-phosphate synthase [UDP-forming] 1-like (LOC100797320), transcript variant X6, mRNA	3542	0.12
		*Glyma.15G244000.1*	3PREDICTED: Glycine max uncharacterized LOC100814749 (LOC100814749), mRNA	1213	0.10
		*Glyma.15G261100.1*	3PREDICTED: Glycine max uncharacterized LOC100801946 (LOC100801946), transcript variant X1, mRNA	3888	0.09
2019	Chr19	*Glyma.19G110600.1*	3PREDICTED: Glycine max uncharacterized LOC102659858 (LOC102659858), mRNA	1709	0.10
	Chr02	*Glyma.02G220300.1*	2PREDICTED: Glycine max ataxin-2-like (LOC100788042), mRNA	1135	0.18
	Chr04	*Glyma.04G102900.1*		2522	0.10
	Chr08	*Glyma.08G071600.1*	2PREDICTED: Glycine max metacaspase-3-like (LOC100796113), transcript variant X2, mRNA	1839	0.12
	Chr11	*Glyma.11G229600.1*	3PREDICTED: Glycine max DNA replication complex BAG protein, transcript variant 2, mRNA,	1257	0.47
	Chr12	*Glyma.12G224000.1*	2PREDICTED: Glycine max uncharacterized LOC102660202 (LOC102660202), mRNA	2799	0.17
		*Glyma.12G227300.1*	2PREDICTED: Glycine max DNA ligase 1-like (LOC100818049), mRNA	2728	0.17
	Chr20	*Glyma.20G026100.1*	2PREDICTED: Glycine max 26S proteasome non-ATPase regulatory subunit 7 homolog A-like (LOC100816479), mRNA	896	0.08

### Expression of two candidate genes in different tissues

**Fig. 8:**
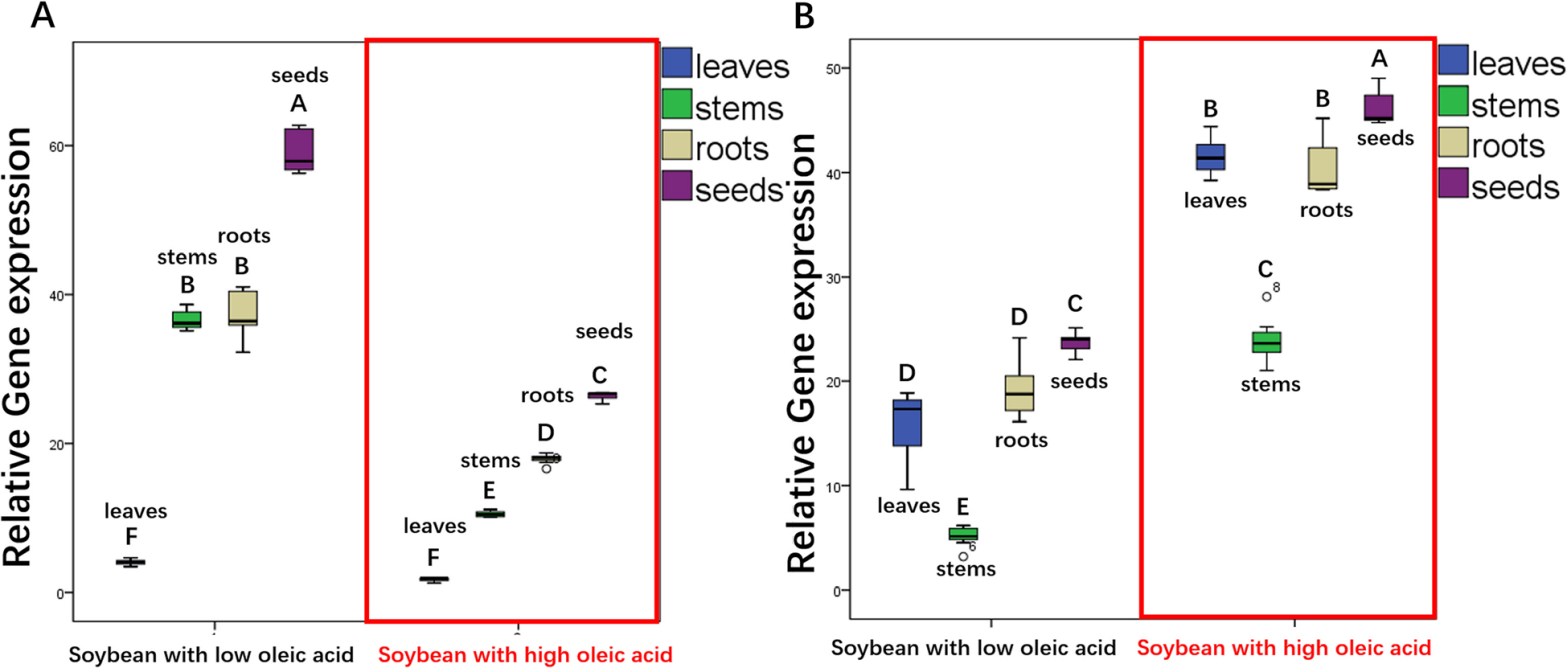
Box-plot representation of two candidate genes expression in different tissues. **a** Variance analysis of *Glyma.11G229600.1* expression in different tissues of soybean. **b** Variance analysis of *Glyma.04G102900.1* expression in different tissues of soybean. The different uppercase letters indicate significant differences at *P* < 0.01, as determined by Duncan’s multiple-range test

To validate their association with oleic acid content, the expression of the two selected candidate genes was measured in different tissues (root, stem, leaf and seed) by using qRT-PCR. The *lectin* gene (GenBank: A5547–127) was used as the reference gene. The results showed that the candidate gene *Glyma.11G229600.1* in soybean seedlings was expressed in different tissues, but the relative expression level of the gene was differed significantly, ranging from 1.23 to 4.31 in soybean leaves, from 10.21 to 39.56 in stems, and from 16.21 to 43.14 in roots (Fig. 8a). The candidate gene *Glyma.11G229600.1* had the lowest relative expression level (1.23) in the leaves of the soybean line q001, which has the lowest oleic acid content. The relative expression level of *Glyma.11G229600.1* in the seeds of the soybean line q001 was also the lowest (25.26). The relative expression level of the candidate gene *Glyma.11G229600.1* in the leaves of the soybean line q353 was 4.3 times higher than that in the leaves of the soybean line q001, and the level in seeds was 10 times higher than that in leaves (Additional file 5: Fig. S3). In general, the correlation coefficient between *Glyma.11G229600.1* and the oleic acid content was 0.980–0.994 (*P<0.01*) (Additional file 6: Table. S3). This result strongly indicates that the candidate gene *Glyma.11G229600.1* plays a positive role in regulating the oleic acid content in seeds.

The relative expression of *Glyma.04G102900.1* was also analysed in soybean leaves, stems, roots and seeds. The relative expression level of *Glyma.04G102900.1* in leaves ranged from 9.62 to 44.41 and that in stems ranged from 3.18 to 28.11 (Fig. 8b). Specifically, in the soybean line q001, which has the lowest oleic acid content in seeds, the candidate gene *Glyma.04G102900.1* showed the highest expression level, which was 49.01 (Additional file 7: Fig. S4). In general, the relative expression level of *Glyma.04G102900.1* in different tissues differed significantly (*P<0.05*), with a correlation coefficient between *Glyma.04G102900.1* and oleic acid content of − 0.964 ~ − 0.998 (Additional file 8: Table. S4). This result indicates that *Glyma.04G102900.1* is closely related to the oleic acid content, specifically showing a negative effect on the oleic acid content in seeds.

## Discussion

Soybean oil is composed of five fatty acids: palmitic acid (16:0), stearic acid (18:0), oleic acid (18:1), linoleic acid (18:2), and linolenic acid (18:3) [17], and the percentage of these five fatty acids in soybean oil averages 10, 4, 18, 55, and 13%, respectively. It was found that the oleic acid content in grains of different soybean varieties varied greatly [18]. Japanese scientists collected 319 Japanese soybean varieties in 2016; among the 319 accessions, the oleic acid content in seeds ranged from 7.66 to 15.86%, and 101 accessions had seed oleic acid levels of 11.5% [19]. In 2008, cultivated and wild soybean germplasms from different regions were analysed for their fatty acid content. The results showed that the average fat content in cultivated soybean was 17.21%, which was 6.22% higher than that in wild soybean; the oleic acid content in cultivated soybean was 23.25%, which was 7.75% higher than that in wild soybean; and the linoleic acid content was 53.53%, which was 2.57% lower than that in wild soybean [20]. In this study, the oleic acid content ranged from 13.5 to 38.4%. The results also showed that the average oleic acid content in soybean germplasms varied greatly, and the oleic acid content in soybean grain was significantly different between regions. In Kurt’s study, the correlation analysis clearly indicated a significant and negative correlation of oleic acid with linoleic acid (*r* = − 0.701, *P* < 0.0001) and stearic acid (*r* = − 0.218, *P* < 0.001), stearic acid was significantly positively correlated with oleic and arachidic acid, while stearic acid had an inverse association with both linoleic and linolenic acids [21]. In our study, the oleic acid content was also significantly positively correlated with linoleic acid (0.454). The results indicated that the relationship between oleic and linoleic acids may be helpful in evaluating varieties that are rich in oleic acid.

*Glyma.11G229600.1,* located on chromosome 11, was simultaneously detected by GWAS during 2018 and 2017. *Glyma.11G229600.1* belongs to the plant BAG protein family. The BAG proteins are a broadly conserved gene family with homologs spanning wide evolutionary distances, including yeast, animals, and plants [22]. Studies have shown that BAG proteins are also present in *Arabidopsis thaliana* [23]. The BAG protein family plays an important role in plant growth and development. Overexpression of BAG7 can increase plant sensitivity to temperature, and BAG4 encodes anti-apoptotic genes that have been reported to confer tolerance to salinity and drought stresses in transgenic tobacco [24]. Drought treatment at different growth stages also contributed to differences in fatty acids [25]. The fatty acid composition and amino acid composition were significantly affected by drought stress [26]. Severe drought increased the protein content by 4.4 percentage points, while the oil content decreased by 2.9 percentage points. With increasing drought stress, measured as accumulating stress over days, the protein content increased linearly, and the oil content decreased [27]. It can be speculated that *Glyma.11G229600.1* may increase the drought tolerance of soybean, thus affecting the accumulation of oleic acid in soybean seeds.

*Glyma.04G102900.1* belongs to the plant GRAS protein family. GRAS proteins constitute an important family of plant-specific proteins named after the first three members discovered: gibberellic acid insensitive (GAI), repressor of gai (RGA) and scarecrow (SCR). At least 33 GRAS genes have been identified in *A. thaliana* and rice [28]. Two GRAS domain proteins have recently been discovered in pulses [29]. Rhizobial bacteria enter a symbiotic interaction with pulses, activating diverse responses in roots through the lipochito oligosaccharide signalling molecule Nod factor. Indeed, a study showed that a GRAS protein transduces calcium signals in plants and acts as a possible regulator of Nod-factor-inducible gene expression [30]. In this study, we investigated the expression of two candidate genes in different independent soybean lines by qRT-PCR and found that the expression of the candidate genes varied in these lines. We discovered two genes that were correlated with oleic acid content in soybean seed in both the 2018 and 2019 analyses. This is the first time that the key genes *Glyma.04G102900.1* and *Glyma.11G229600.1* have been reported to be associated with the oleic acid content. Hence, further studies should be conducted to support this finding. Our results provide a basis for deciphering the mechanism underlying the determination of fatty acid composition in soybean. Moreover, the SNP markers identified here demonstrate that marker-assisted selection is a powerful strategy for identifying genes of interest in soybean and can be used in breeding programmes aimed at optimizing fatty acid profiles in seeds.

## Conclusions

In this study, the genome-wide association study (GWAS) technique was used to find SNP markers correlated with oleic acid content. In 2018, 20 new candidate genes related to oleic acid content were detected, and in 2018, a total of 8 new candidate genes related to oleic acid content were also detected. *Glyma.04G102900.1* on chromosome 4 and *Glyma.11G229600.1* on chromosome 11 were detected in both analyses (2018 and 2019). *Glyma.04G102900.1* and *Glyma.11G229600.1* are new key candidate genes related to oleic acid in soybean seeds.

## Methods

### Plant materials

The 260 soybean materials provided by the Biotechnology Center of Jilin Agricultural University were planted in the experimental field of Jilin Agricultural University (Changchun, China) from 2018 to 2019 (total of 2 years). A randomized complete block design was used. Each soybean line was examined using three biological replicates. The field was divided into three blocks of 260 m^2^ (26 × 10 m), and each block was subdivided into eight sections. Each section was subdivided into 260 subsections. Natural drying (sunlight) was allowed to occur, and then, the seeds were threshed for oleic acid determination. Fourteen soybean varieties with significantly different oleic acid levels were selected to test candidate gene expression. The names of the soybean lines and the fatty acid content are shown in Table 3.

**Table 3:** Names of 14 soybean lines and average levels of five fatty acids

Group	Name of line	Oil	Protein	Oleic acid	Linoleic acid	Linolenic acid	Palmitic acid	Stearic acid
Low oleic acid content	q001	21.1	37.62	9.56	67.85	10.77	9.79	1.26
	q024	20.18	39.1	10.42	67.19	8.57	9.02	0.75
	q020	20.94	39.12	11.55	65.56	8.6	8.59	1.01
	q008	17.94	43.49	11.67	64.32	7.75	9.48	1.01
	q035	19.13	39.17	11.99	62.45	9.43	8.73	1.38
	T7287	20.96	37.76	12.23	60.62	7.74	8.22	2.36
	q033	21.1	38.03	12.4	62.92	9.33	9.86	0.81
High oleic acid content	q318	16.88	40.29	25.34	57.71	8.17	2.32	3.27
	q20	22.19	40.42	26.41	54.1	6.04	2.65	1.8
	q073	21.29	41.54	28.08	50.94	7.97	1.72	2.69
	q176	12.36	38.91	29.73	55.13	8.85	0.76	5.06
	q070	20.9	40.99	29.78	51.08	7.09	1.67	1.89
	q68	16.91	39.44	29.94	53.64	6.01	0.56	4.43
	q353	15.59	39.49	31.02	52.18	8.6	0.99	5.73

### Determination of fatty acid levels in soybean seeds

The levels of oleic acid and four other fatty acids (stearic acid, palmitic acid, linoleic acid and linolenic acid) in soybean seeds were determined by a NIRSTM DS 2500 instrument (FOSS, Hillerod, Denmark) after harvesting. SPSS version 22.0 software (SPSS Inc., Chicago, IL, USA) was used to calculate the correlation coefficients of fatty acids in soybean seeds.

### Genotyping of soybean germplasms

Total genomic DNA was extracted from the leaves of each soybean line using the CTAB method according to Murray & Thompson (1980) [31]. The 260 soybean materials were genotyped by specific-locus amplified fragment sequencing (SLAF-seq), and SNP molecular markers were developed. DNA extraction is the first step in sequencing. SNP molecular markers are used for phylogenetic analysis and genetic evolutionary correlation analysis. The restriction endonuclease combination was *RsaI-HaeIII*. The sequencing service was provided by Beijing Biomarker Biotechnology Company, PR China.

### Population structure evaluation

Principal component analysis (PCA) was used to assess the population structure using the EIGENSOFT software package. Based on the neighbour-joining method, MEGA5 software was used to construct a phylogenetic tree that included each sample.

### Genome-wide association study (GWAS)

Based on the SNP markers obtained by SLAF-Seq technology, the correlation values between SNP markers and oleic acid content were obtained by using the five models (glm, mlm, cmlm, fastlmm and emmax) in TASSEL software. TASSEL software can calculate the Q matrix of the sample population structure according to the *K* matrix and finally obtain a correlation value for each SNP marker. The results of each model of each trait were annotated based on a 0.000001 level of significance. In this experiment, both the Manhattan map and QQ map were constructed using Haploview software. The Manhattan map was used to represent the correlation between genotype data and phenotypic data. The QQ map was used to represent the level of difference between observed and predicted values. In this study, the candidate genes were predicted by using the Swiss-Prot and NR databases.

### Quantitative reverse transcription PCR

qRT-PCR analysis was performed using a Bio-Rad CFX system (Amersham Biosciences, Little Chalfont, Buckinghamshire, UK). Total RNA was extracted using an Eastep® Super Total RNA Extraction Kit (TaKaRa Company, Kusatsu, Shiga, Japan). The amplification reaction conditions were as follows: predenaturation at 95 °C for 10 min, denaturation at 95 °C for 10 s, followed by annealing at 53 °C for 20 s, and extension at 72 °C for 15 s. The amplification reaction conditions for the *Glyma.11G229600.1* gene were as follows: 95 °C for 10 min; 35 cycles of denaturation at 95 °C for 30 s, annealing at 67 °C for 30 s, and extension at 72 °C for 30 s; and extension at 72 °C for 10 min. All of the above reactions included 40 cycles. After amplification, the dissolution curve was calculated by the 2^-ΔΔCt^ method [32]. The amplification reaction conditions for the gene *Glyma.04G102900.1* were as follows: predenaturation at 95 °C for 10 min; 35 cycles of denaturation at 95 °C for 30 s, annealing at 59 °C for 30 s, and extension at 72 °C for 35 s; and extension at 72 °C for 10 min. Three biological replicates were used for each gene.

### Data analysis

The phenotypic data were measured and recorded using Microsoft Excel 2010 software. Differential saliency analysis, analysis of variance, correlation analysis and descriptiveness analysis were performed by using SPSS 19.0 (IBM Corp, Armonk, NY, USA) software [33]. The positive and negative maps and histograms were constructed by using GraphPad Prism software (GraphPad Company, San Diego, CA).

## Supplementary information

**Additional file 1 Table S1.** Heritability of fatty acid content in soybean seeds.

**Additional file 2 Figure S1.** Population structure of the soybean germplasm collection. (PPTX 182 kb)

**Additional file 3 Table S2.** SNPs identified as being associated with oleic acid content in 2 years (*P* < 0.000001).

**Additional file 4 Figure S2.** Genome-wide linkage disequilibrium (LD) decay for all 260 accessions. (PPTX 223 kb)

**Additional file 5 Figure S3.** Expression of *Glyma.11G229600.1* in different tissues of soybean lines. (PPTX 354 kb)

**Additional file 6 Table S3.** Correlation between *Glyma.11G229600.1* expression and oleic acid content.

**Additional file 7 Figure S4.** Expression of *Glyma.04G102900.11* in different tissues of soybean lines. (PPTX 233 kb)

**Additional file 8 Table S4** Correlation between *Glyma.04G102900.1* expression and oleic acid content.

## Data Availability

The raw sequence information that support the findings of this study are available from [Beijing Biomarker Biotechnology Co., ltd, Beijing, China] but restrictions apply to the availability of these data, which were used under license for the current study, and so are not publicly available. Data are however available from the corresponding authors upon reasonable request and with permission of [Beijing Biomarker Biotechnology Co., ltd, Beijing, China]. Other datasets supporting the conclusions of this article are included within the article and its additional files.

## Abbreviations

SNP: Single-nucleotide polymorphism
SLAF-seq: Specific-locus amplified fragment sequencing
GWAS: Genome-wide association study
LD: Linkage disequilibrium
qRT-PCR: Real-time quantitative PCR
cM: Centimorgan
QTL: Quantitative trait locus
PCA: Principal component analysis
QQ: Quantile-quantile
