# Supplementary material for: Genome-wide association study identifies candidate genes related to oleic acid content in soybean seeds
Source: BMC Plant Biol. 2020 Aug 28;20:399. doi: 10.1186/s12870-020-02607-w (PMC7456086; doi:10.1186/s12870-020-02607-w)
Supplement: Supplementary file 1 — Additional file 1 Table S1. Heritability of fatty acid content in soybean seeds. [file 12870_2020_2607_MOESM1_ESM.pdf]

Table S1 Heritability of fatty acid content in soybean seeds

Traits	oleic acid	linoleic acid	linolenic acid	stearic acid	palmitic acid
Heritability	0.653	0.423	0.403	0.253	0.208
δgl^2	184.1	220.1	47.1	89.3	62.1
δe^2	92.5	45.4	9.5	18.2	21.1

Note: H^2: Broad sense heritability, δe^2: Error variance, δgl^2: Gene and environment interaction variance.
