# Supplementary material for: Genome-wide association study identifies candidate genes related to oleic acid content in soybean seeds
Source: BMC Plant Biol. 2020 Aug 28;20:399. doi: 10.1186/s12870-020-02607-w (PMC7456086; doi:10.1186/s12870-020-02607-w)
Supplement: Supplementary file 2 — Additional file 2 Figure S1. Population structure of the soybean germplasm collection. (PPTX 182 kb) [file 12870_2020_2607_MOESM2_ESM.pptx]

## Slide 1
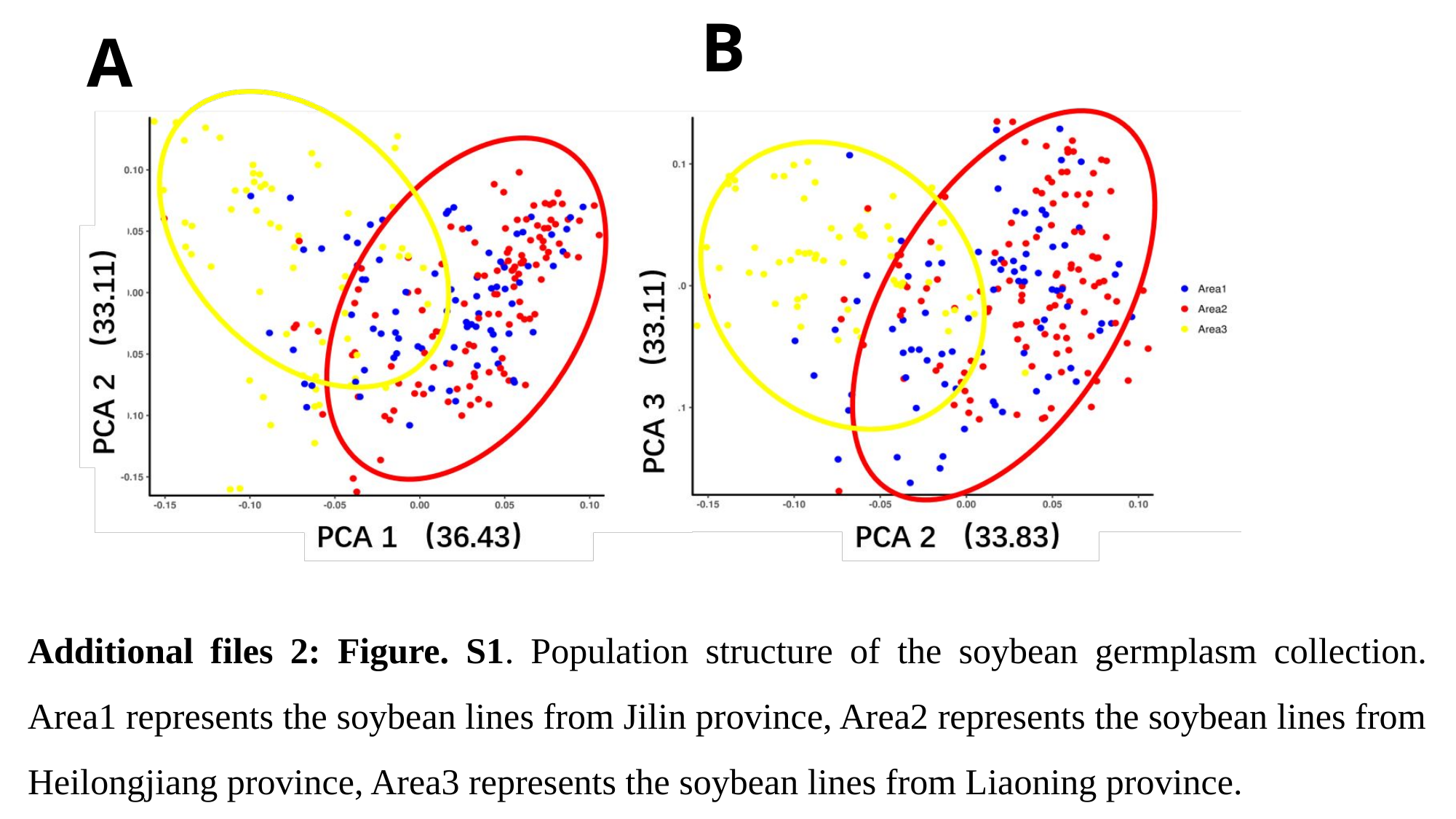

B
A
Additional files 2: Figure. S1. Population structure of the soybean germplasm collection. Area1 represents the soybean lines from Jilin province, Area2 represents the soybean lines from Heilongjiang province, Area3 represents the soybean lines from Liaoning province.
