# Supplementary material for: Genome-wide association study identifies candidate genes related to oleic acid content in soybean seeds
Source: BMC Plant Biol. 2020 Aug 28;20:399. doi: 10.1186/s12870-020-02607-w (PMC7456086; doi:10.1186/s12870-020-02607-w)
Supplement: Supplementary file 3 — Additional file 3 Table S2. SNPs identified as being associated with oleic acid content in 2 years (P < 0.000001). [file 12870_2020_2607_MOESM3_ESM.pdf]

Table S2 SNPs identified to be associated with oleic acid content in two years (P < 0.000001)

Year	traits	Model	Chr	Location	P value	log10P	MarkerR2	Allele	Count	MAF
2018	Oleic acid	cmlm	Chr04	21863261	1.34E-08	7.87	0.27167	C/T	C:257_T:17	0.06
	Oleic acid	cmlm	Chr12	21089058	2.36E-08	7.63	0.19868	C/G	C:297_G:35	0.11
	Oleic acid	cmlm	Chr12	21089219	2.63E-08	7.58	0.19921	A/G	A:297_G:35	0.11
	Oleic acid	cmlm	Chr15	14988985	2.59E-09	8.59	0.28042	C/G	C:142_G:120	0.46
	Oleic acid	cmlm	Chr15	14989032	1.84E-09	8.74	0.28124	T/G	T:142_G:124	0.47
	Oleic acid	emmax	Chr04	21863261	1.65E-07	6.78	--	C/T	C:257_T:17	0.06
	Oleic acid	fastlmm	Chr04	21863261	1.55E-07	6.81	--	C/T	C:257_T:17	0.06
	Oleic acid	mlm	Chr04	21863261	8.17E-09	8.09	0.2682	C/T	C:257_T:17	0.06
	Oleic acid	mlm	Chr12	21089058	5.79E-09	8.24	0.20735	C/G	C:297_G:35	0.11
	Oleic acid	mlm	Chr12	21089219	6.65E-09	8.18	0.20726	A/G	A:297_G:35	0.11
	Oleic acid	mlm	Chr15	14988985	6.17E-10	9.21	0.28935	C/G	C:142_G:120	0.46

Oleic acid	mlm	Chr15	14989032	4.88E-10	9.31	0.28773	T/G	T:142_G:124	0.47
Oleic acid	mlm	Chr19	48048543	3.03E-08	7.52	0.20318	C/A	C:165_A:139	0.46
Oleic acid	glm	Chr01	39607851	1.27E-09	8.9	0.21036	G/A	G:221_A:45	0.17
Oleic acid	glm	Chr01	48111227	1E-08	8	0.15183	G/T	G:353_T:37	0.09
Oleic acid	glm	Chr04	21863261	4.52E-10	9.34	0.25254	C/T	C:257_T:17	0.06
Oleic acid	glm	Chr04	23790905	1.98E-08	7.7	0.10936	C/T	C:473_T:29	0.06
Oleic acid	glm	Chr04	23790963	1.98E-08	7.7	0.10936	C/A	C:473_A:29	0.06
Oleic acid	glm	Chr04	24348133	5.37E-08	7.27	0.11388	T/C	T:413_C:31	0.07
Oleic acid	glm	Chr04	41082237	1.74E-09	8.76	0.16959	A/C	A:343_C:21	0.06
Oleic acid	glm	Chr06	35774028	4.09E-08	7.39	0.15766	C/T	C:293_T:23	0.07
Oleic acid	glm	Chr13	6470698	1.32E-08	7.88	0.19166	A/C	A:270_C:20	0.07
Oleic acid	glm	Chr13	12857385	2.42E-08	7.62	0.13167	T/A	T:259_A:147	0.36

Oleic acid	glm	Chr14	48228305	4.28E-08	7.37	0.13476	G/A	G:374_A:30	0.07
Oleic acid	glm	Chr15	14988985	3.94E-11	10.4	0.2716	C/G	C:142_G:120	0.46

Year	traits	model	chr	Location	P-value	log10P	MarkerR2	Allele	Count	MA[illegible]
2019	Oleic acid	cmlm	Chr03	6843120	2.08E-08	7.68	0.17373	A/T	A:441_T:29	0.06
	Oleic acid	cmlm	Chr04	19175418	2.17E-08	7.66	0.18449	G/A	G:323_A:29	0.08
	Oleic acid	cmlm	Chr06	30182914	3.65E-08	7.44	0.24762	A/G	A:237_G:35	0.13
	Oleic acid	cmlm	Chr10	13263622	3.87E-08	7.41	0.23712	G/A	G:227_A:71	0.24
	Oleic acid	cmlm	Chr18	3181501	4.69E-08	7.33	0.26556	T/C	T:209_C:53	0.2
	Oleic acid	cmlm	Chr19	16522609	4.97E-08	7.3	0.22756	C/T	C:306_T:22	0.07
	Oleic acid	cmlm	Chr20	38714788	1.14E-08	7.94	0.16419	G/A	G:437_A:23	0.05
	Oleic acid	cmlm	Chr20	38714828	1.18E-08	7.93	0.16415	G/A	G:437_A:23	0.05
	Oleic acid	emmax	Chr03	32904059	2.43E-07	6.61	--	G/T	G:469_T:25	0.05
	Oleic acid	fastlmm	Chr03	32904059	2.16E-07	6.67	--	G/T	G:469_T:25	0.05

Oleic acid	glm	Chr03	6843120	6.95E-11	10.16	0.16138	A/T	A:441_T:29	0.06
Oleic acid	glm	Chr03	11643875	2.02E-09	8.69	0.25207	A/G	A:207_G:65	0.24
Oleic acid	glm	Chr04	19175418	2.96E-08	7.53	0.1719	G/A	G:323_A:29	0.08
Oleic acid	glm	Chr06	30182914	4.64E-09	8.33	0.24411	A/G	A:237_G:35	0.13
Oleic acid	glm	Chr08	42380726	7.71E-11	10.11	0.28375	A/G	A:211_G:55	0.21
Oleic acid	glm	Chr08	42381023	5.93E-11	10.23	0.28514	G/A	G:213_A:55	0.21
Oleic acid	glm	Chr10	13263622	4.25E-09	8.37	0.22409	G/A	G:227_A:71	0.24
Oleic acid	glm	Chr11	175043	5.4E-11	10.27	0.2324	T/A	T:259_A:69	0.21
Oleic acid	glm	Chr15	31819904	1.33E-10	9.88	0.26587	C/T	C:211_T:73	0.26
Oleic acid	glm	Chr19	16522609	4.23E-10	9.37	0.21528	C/T	C:306_T:22	0.07
Oleic acid	glm	Chr20	38714788	5.17E-10	9.29	0.15974	G/A	G:437_A:23	0.05
