# Supplementary material for: Genome-wide association study identifies candidate genes related to oleic acid content in soybean seeds
Source: BMC Plant Biol. 2020 Aug 28;20:399. doi: 10.1186/s12870-020-02607-w (PMC7456086; doi:10.1186/s12870-020-02607-w)
Supplement: Supplementary file 4 — Additional file 4 Figure S2. Genome-wide linkage disequilibrium (LD) decay for all 260 accessions. (PPTX 223 kb) [file 12870_2020_2607_MOESM4_ESM.pptx]

## Slide 1
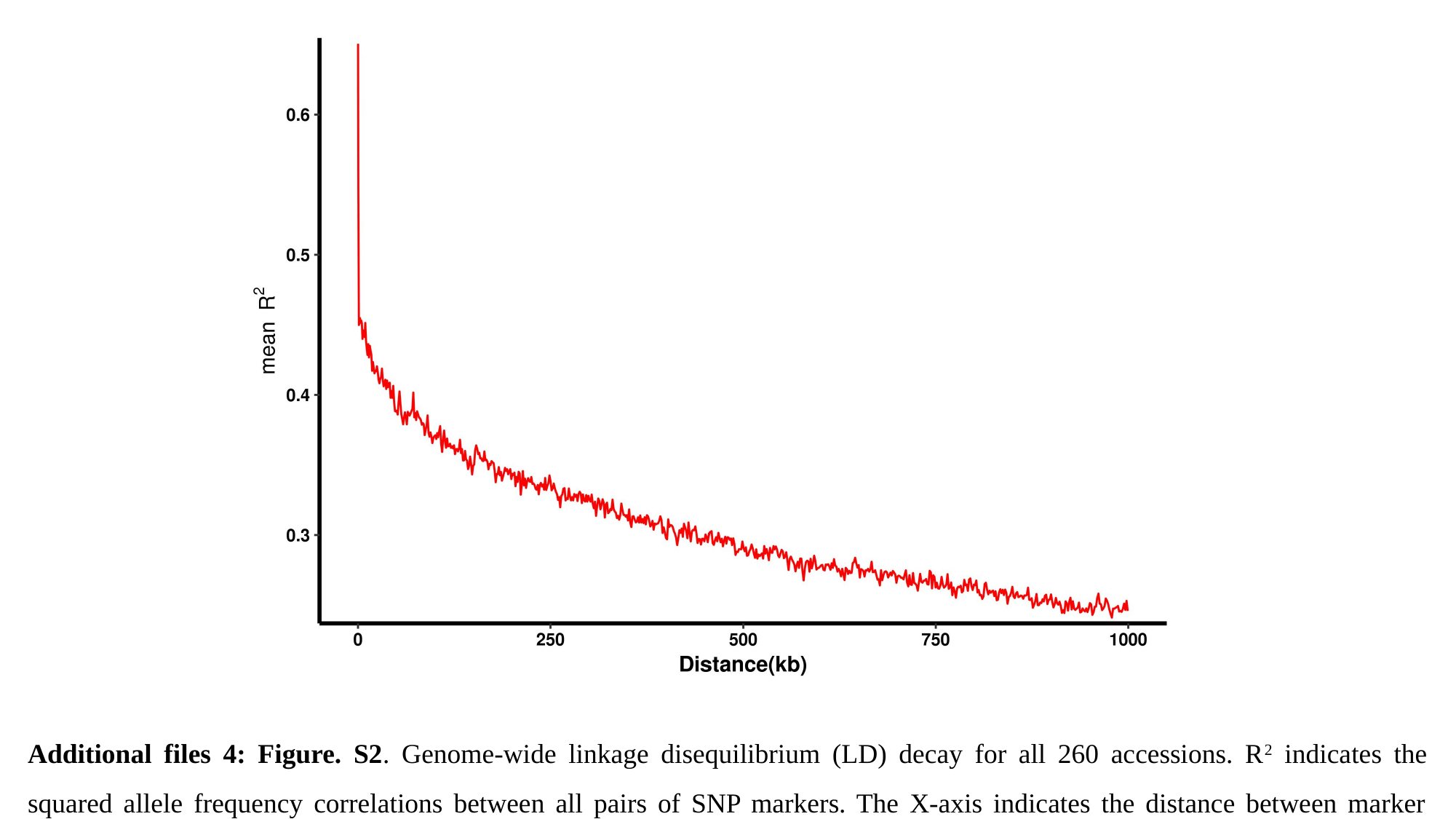

Additional files 4: Figure. S2. Genome-wide linkage disequilibrium (LD) decay for all 260 accessions. R2 indicates the squared allele frequency correlations between all pairs of SNP markers. The X-axis indicates the distance between marker pairs.
