# Supplementary material for: Genome-wide association study identifies candidate genes related to oleic acid content in soybean seeds
Source: BMC Plant Biol. 2020 Aug 28;20:399. doi: 10.1186/s12870-020-02607-w (PMC7456086; doi:10.1186/s12870-020-02607-w)
Supplement: Supplementary file 5 — Additional file 5 Figure S3. Expression of Glyma.11G229600.1 in different tissues of soybean lines. (PPTX 354 kb) [file 12870_2020_2607_MOESM5_ESM.pptx]

## Slide 1
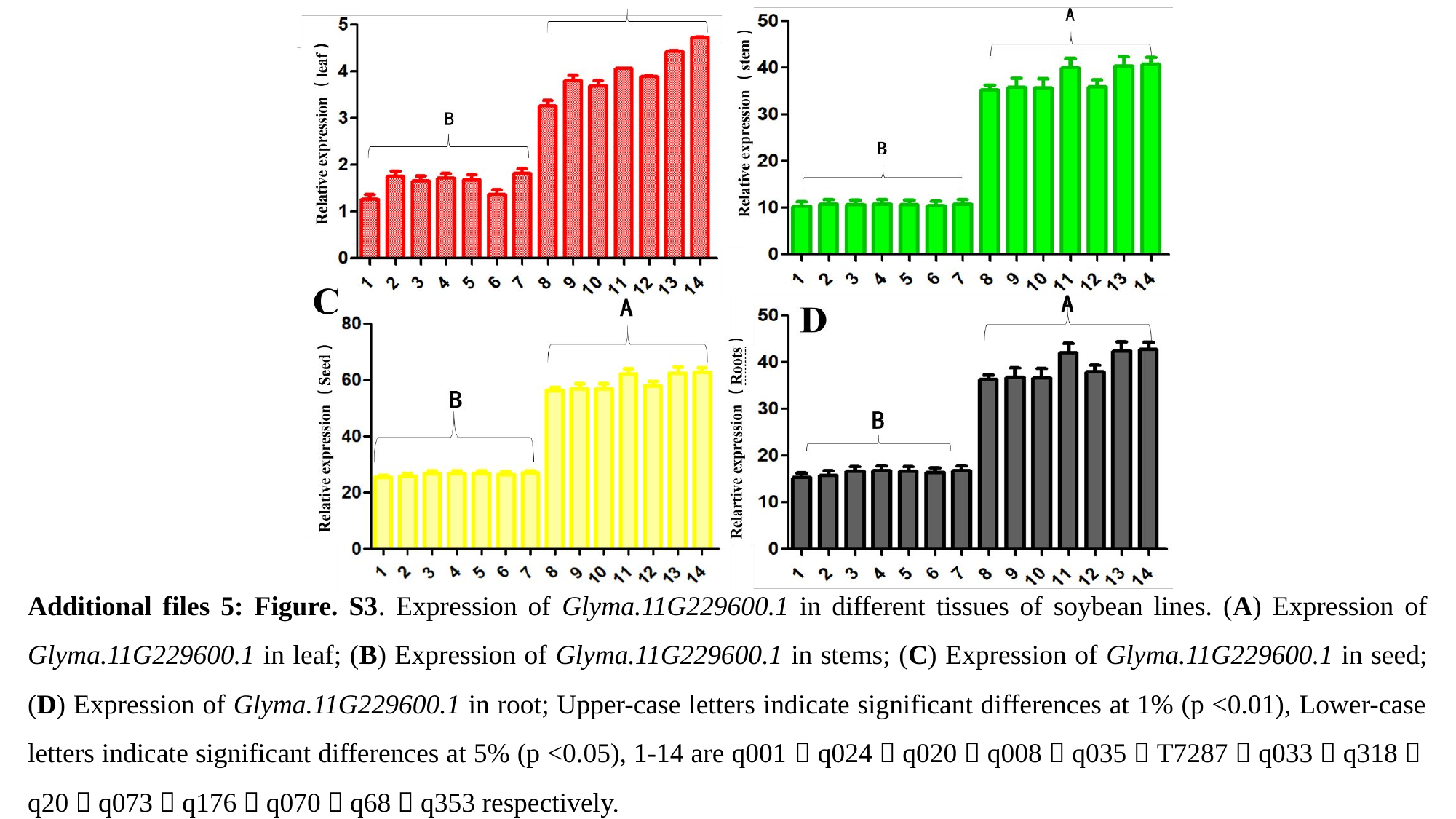

Additional files 5: Figure. S3. Expression of Glyma.11G229600.1 in different tissues of soybean lines. (A) Expression of Glyma.11G229600.1 in leaf; (B) Expression of Glyma.11G229600.1 in stems; (C) Expression of Glyma.11G229600.1 in seed; (D) Expression of Glyma.11G229600.1 in root; Upper-case letters indicate significant differences at 1% (p <0.01), Lower-case letters indicate significant differences at 5% (p <0.05), 1-14 are q001，q024，q020，q008，q035，T7287，q033，q318，q20，q073，q176，q070，q68，q353 respectively.
