# Supplementary material for: Genome-wide association study identifies candidate genes related to oleic acid content in soybean seeds
Source: BMC Plant Biol. 2020 Aug 28;20:399. doi: 10.1186/s12870-020-02607-w (PMC7456086; doi:10.1186/s12870-020-02607-w)
Supplement: Supplementary file 6 — Additional file 6 Table S3. Correlation between Glyma.11G229600.1 expression and oleic acid content. [file 12870_2020_2607_MOESM6_ESM.pdf]

Table S3 Correlation between the *Glyma.11G229600.1* expression and oleic acid content

		Oleic acid content	Relative expression in leaves	Relative expression in stems	Relative expression in roots	Relative expression in seeds
Oleic acid content	Pearson relative	1	0.980**	0.984**	0.990**	0.994**
Relative expression in leaves	Pearson relative	0.980**	1	0.986**	0.977**	0
Relative expression in stems	Pearson relative	0.984**	0.986**	1	0.987**	0.994**
Relative expression in roots	Pearson relative	0.990**	0.977**	0.987**	1	0.994**
Relative expression in seeds	Pearson relative	0.994**	0.981**	0.998**	0.994**	1

Note: The '**' indicate significant differences at $P < 0.01$, The '*' indicate significant differences at $P < 0.05$, as determined by Duncan's multiple-range test.
