# Supplementary material for: Genome-wide association study identifies candidate genes related to oleic acid content in soybean seeds
Source: BMC Plant Biol. 2020 Aug 28;20:399. doi: 10.1186/s12870-020-02607-w (PMC7456086; doi:10.1186/s12870-020-02607-w)
Supplement: Supplementary file 7 — Additional file 7 Figure S4. Expression of Glyma.04G102900.11 in different tissues of soybean lines. (PPTX 233 kb) [file 12870_2020_2607_MOESM7_ESM.pptx]

## Slide 1
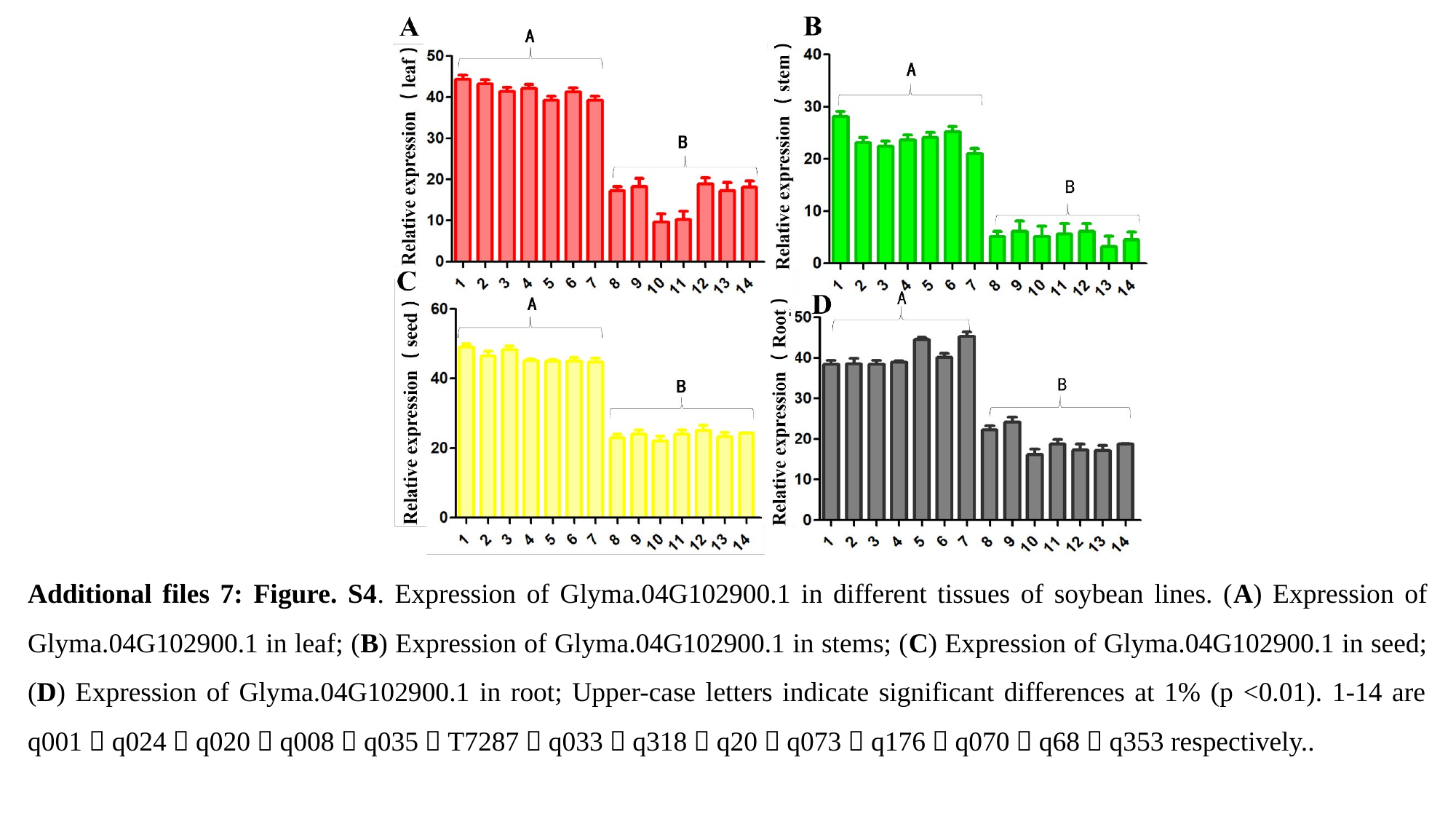

Additional files 7: Figure. S4. Expression of Glyma.04G102900.1 in different tissues of soybean lines. (A) Expression of Glyma.04G102900.1 in leaf; (B) Expression of Glyma.04G102900.1 in stems; (C) Expression of Glyma.04G102900.1 in seed; (D) Expression of Glyma.04G102900.1 in root; Upper-case letters indicate significant differences at 1% (p <0.01). 1-14 are q001，q024，q020，q008，q035，T7287，q033，q318，q20，q073，q176，q070，q68，q353 respectively..
