# Supplementary material for: Genome-wide association study identifies candidate genes related to oleic acid content in soybean seeds
Source: BMC Plant Biol. 2020 Aug 28;20:399. doi: 10.1186/s12870-020-02607-w (PMC7456086; doi:10.1186/s12870-020-02607-w)
Supplement: Supplementary file 8 — Additional file 8 Table S4 Correlation between Glyma.04G102900.1 expression and oleic acid content. [file 12870_2020_2607_MOESM8_ESM.pdf]

Table S4 Correlation between *Glyma.04G102900.1* expression and oleic acid content

		Oleic acid content	Relative expression in leaves	Relative expression in stems	Relative expression in roots	Relative expression in seeds
Oleic acid content	Pearson relative	1	-0.969**	-0.982**	-0.964**	0.998**
Relative expression in leaves	Pearson relative	-0.969**	1	0.971**	0.944**	0.998**
Relative expression in stems	Pearson relative	-0.982**	0.971**	1	0.947**	0.998**
Relative expression in roots	Pearson relative	-0.964**	0.944**	0.947**	1	0.998**
Relative expression in seeds	Pearson relative	-0.998**	0.945**	0.923**	0.911**	1

Note: '**' indicate significant differences at $P < 0.01$, The '*' indicate significant differences at $P < 0.05$, as determined by Duncan's multiple-range test.
